# Machine learning for predicting the risk stratification of 1–5 cm gastric gastrointestinal stromal tumors based on CT

**DOI:** 10.1186/s12880-023-01053-y

**Published:** 2023-07-06

**Authors:** Cui Zhang, Jian Wang, Yang Yang, Bailing Dai, Zhihua Xu, Fangmei Zhu, Huajun Yu

**Affiliations:** 1 Department of Radiology, TongDe Hospital of Zhejiang Province, No.234, Gucui Road, Hangzhou, Zhejiang China; 2 Department of Radiology, The First Affiliated Hospital of Bengbu Medical College, No.287, Changhuai Road, Bengbu, Anhui China; 3 Department of Radiology, Zhejiang Hospital, No. 12, Lingyin Road, Hangzhou, Zhejiang China

**Keywords:** Gastrointestinal stromal tumors, Machine learning, Risk assessment, X-ray computed tomography

## Abstract

**Backgroud:**

To predict the malignancy of 1–5 cm gastric gastrointestinal stromal tumors (GISTs) by machine learning (ML) on CT images using three models - Logistic Regression (LR), Decision Tree (DT) and Gradient Boosting Decision Tree (GBDT).

**Methods:**

231 patients from Center 1 were randomly assigned into the training cohort (n = 161) and the internal validation cohort (n = 70) in a 7:3 ratio. The other 78 patients from Center 2 served as the external test cohort. Scikit-learn software was used to build three classifiers. The performance of the three models were evaluated by sensitivity, specificity, accuracy, positive predictive value (PPV), negative predictive value (NPV) and area under the curve (AUC). Diagnostic differences between ML models and radiologists were compared in the external test cohort. Important features of LR and GBDT were analyzed and compared.

**Results:**

GBDT outperformed LR and DT with the largest AUC values (0.981 and 0.815) in the training and internal validation cohorts and the greatest accuracy (0.923, 0.833 and 0.844) across all three cohorts. However, LR was found to have the largest AUC value (0.910) in the external test cohort. DT yielded the worst accuracy (0.790 and 0.727) and AUC values (0.803 and 0.700) in both the internal validation cohort and the external test cohort. GBDT and LR performed better than radiologists. Long diameter was demonstrated to be the same and most important CT feature for GBDT and LR.

**Conclusions:**

ML classifiers, especially GBDT and LR with high accuracy and strong robustness, were considered to be promising in risk classification of 1–5 cm gastric GISTs based on CT. Long diameter was found the most important feature for risk stratification.

**Supplementary Information:**

The online version contains supplementary material available at 10.1186/s12880-023-01053-y.

## Backgroud

Gastrointestinal stromal tumors (GISTs) are neoplasms that arise from Cajal cells of the gastrointestinal tract mesenchyme [1]. Nonetheless, they were reported growing throughout the whole digestive tract; stomach was the site with the highest incidence (50–60%) [2]. As a potentially malignant tumor, although less than 2 cm, about 10–30% of GISTs will develop into malignancy, and the risk of recurrence and metastasis is significantly increased [3].

The National Institutes of Health (NIH) classification system has been proposed to stratify the risk of GISTs. Currently, the modified NIH risk stratification criteria and the latest Chinese consensus guidelines (2017 Edition) of the Chinese Society of Clinical Oncology (CSCO) Expert Committee on GIST divide GISTs into very low, low, intermediate, and high risk groups according to tumor size, location, mitotic index, and whether the tumor ruptures [4, 5]. Very low and low risk GISTs generally have slow growth and a low incidence of recurrence and metastasis, whereas GISTs in intermediate and high risk stages have more invasive behavior [6]. Risk classification also facilitates clinical treatment planning [7]. The 2–5 cm GISTs with very low or low risk can be completely resected with endoscopic technology; however, a surgical operation is also necessary in intermediate or high risk ones [8, 9]. Another report suggested periodic follow-up by endoscopic ultrasound (EUS) for GISTs smaller than 1 cm [10]. However, localized GISTs (larger than 1 cm) with intermediate to high risk warranted resection followed by adjuvant treatment of the lesion with imatinib [10–12]. Only 2.2% of gastric GISTs with diameters less than 1 cm have been reported to be considered high-risk, while 1–2 cm GISTs had a malignant risk rate of 10.1% [13]. Most grading systems indicate that GISTs larger than 5 cm have a great tendency for high risk. Therefore, it is clinically meaningful to preoperatively identify high-risk gastric GISTs of 1–5 cm in diameter.

Contrast-enhanced CT (CE-CT) scan can clearly show not only the anatomical structure of gastric mesenchymal tumors, but also the internal and peripheral information of the lesion, including tumor density, necrosis, ulceration, hemorrhage, blood vessels, as well as invasion of surrounding tissues, lymph node metastases, and distant metastases [14, 15].

Plenty of studies predicting the risk stratification of GISTs based on CT imaging have been reported [16–22]. Tumor size was found to be an independent risk factor, even the only one, for high-risk malignant GISTs [16, 21]. Besides, other features such as percentage of tumor necrosis, growth pattern, intratumor angiogenesis, margins, and enhancement pattern were also demonstrated to be contributive to high-grade GISTs on CT images [17, 19, 22].

Machine learning (ML) algorithm provides the possibility of mining valuable data that have significant and intricate connections among enormous data items. ML algorithms have been applied to disease identification, differential diagnosis and prognosis analysis with outstanding performance and promising prospect [23–26]. Most previous studies have used univariate or multivariate logistic regression analysis aiming to predict the malignant potential of GIST. To our knowledge, no research has attempted to classify the risk of GISTs using ML classifier. What’s more, our report focuses on tumors up to 5 cm in the gastric site, which is different from studies including large-sized GISTs in different parts of the gastrointestinal tract. In this study, 309 patients’ CT images of gastric GISTs less than 5 cm were collected to assess the malignancy risk using three models - Logistic Regression (LR), Decision Tree (DT) and Gradient Boosting Decision Tree (GBDT).

## Methods

### Patient selection

**Fig. 1:**
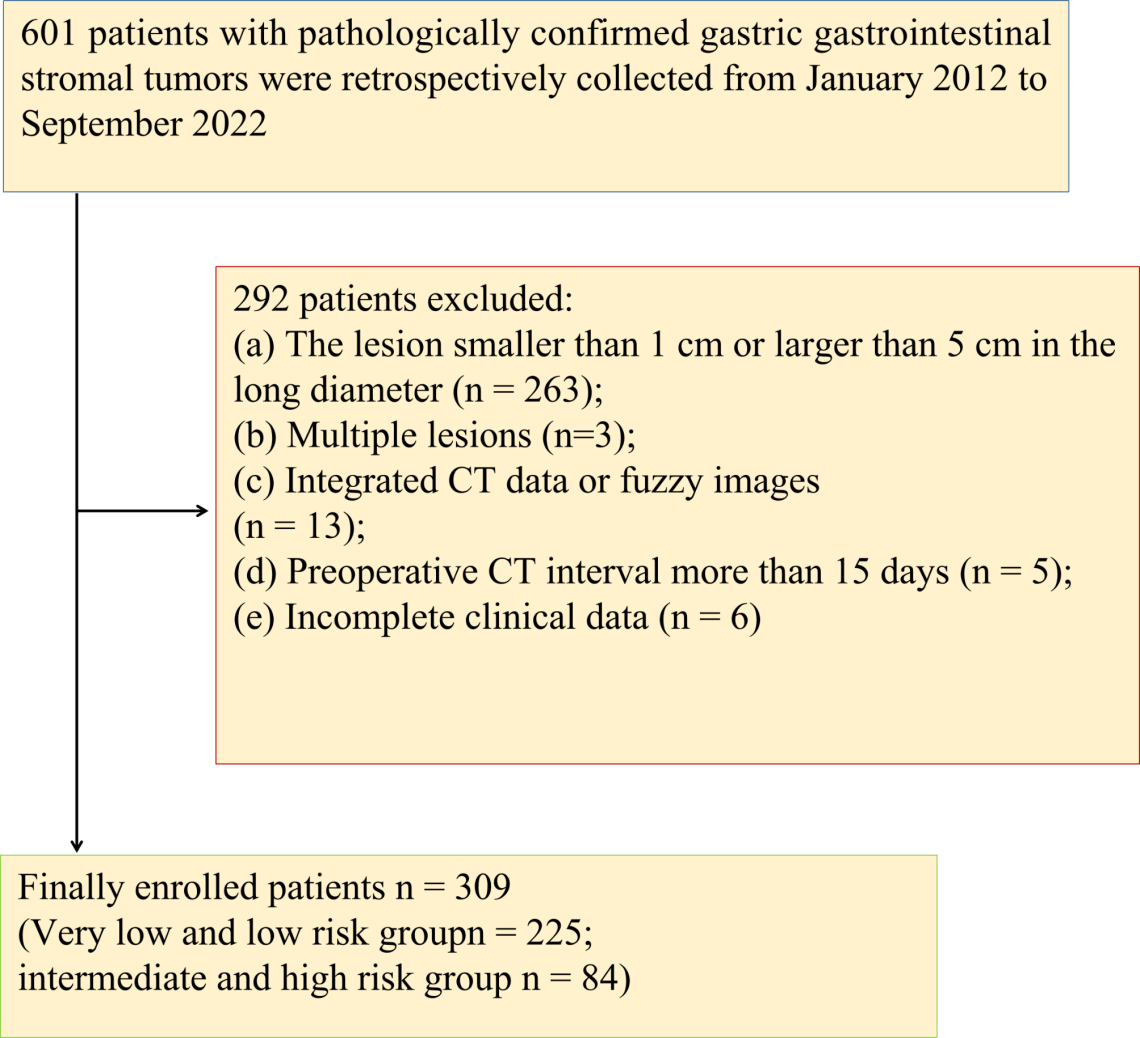
Flowchart shows inclusion and exclusion criteria for this study

This retrospective study was approved by the ethics committee of Tongde Hospital of Zhejiang Province and the need for informed consent was waived (Approval No. 2021-040). Patients with gastric GISTs from two centers (Center 1: Tongde Hospital of Zhejiang Province, Center 2: Zhejiang Hospital) from January, 2012 to September, 2022 were enrolled in this research. The criteria for inclusion were as follows: (a) patients with complete CT images (including unenhanced, arterial and portal venous phase images) within 15 days before surgery; (b) solitary and primary lesions; (c) lesions without neoadjuvant treatment; (d) lesions larger than 1 cm and smaller than 5 cm in the long diameter. (e) patients with detailed clinical data (including age, gender, clinical symptoms and tumor markers). The inclusion and exclusion of patients are shown in Fig. 1. 231 patients from Center 1 were randomly assigned into the training cohort (n = 161) and the internal validation cohort (n = 70) according a 7:3 ratio. Another 78 patients from Center 2 were served as an external test cohort. Clinical characteristics including age, gender, symptoms and tumor markers were collected for each patient. All surgically resected lesions of GISTs were finally divided into a low-grade malignancy group and a high-grade malignancy group. The low-grade malignancy category consisted of GISTs with very low or low risk and the high-grade malignancy group included GISTs with intermediate or high risk. The NIH modified criteria in 2008 [4] were applied for risk stratification.

### CT examination

All patients underwent abdominal CE-CT examination using two 64-slice spiral CT scanners (Siemens, Forchheim, Germany or Philips Medical Systems, Cleveland, OH, USA). The parameters of CT imaging were set as follows: for Siemens, 120 kV tube voltage, 150–250 mA tube current, 0.5 s tube rotation time, 64 × 0.6 mm detector collimation, 350 × 350 mm field of view, 5 mm section thickness and 1-1.25 mm reconstruction interval; for Philips, 120 kV tube voltage, 200–250 mA tube current, 0.5 s tube rotation time, 64 × 0.625 mm detector collimation, 350 × 350 mm field of view, 5 mm section thickness and 1-1.5 mm reconstruction interval. Subsequently, arterial phase (delay 30–40 s) and the portal venous phase (delay 60–70 s) images were obtained after 2 mL/kg of iodinated contrast medium was injected intravenously at a rate of 3 ml/s.

### Image analysis

Two radiologists (Reviewer 1 with 6 years and Reviewer 2 with 13 years of experience in abdominal imaging) independently reviewed CT images and reached final conclusions by consensus without knowledge of the surgical and pathological information of every patient. The determined CT imaging features included (a) the CT attenuation values (Hounsfield units, HU) in unenhancement phase (CTU), (b) in arterial phase (CTA) and (c) in venous phase (CTV) of the tumor, (d) degree of enhancement in arterial phase (DEAP) and (e) in portal venous phase (DEPP), (f) enhanced potentiality in arterial phase (EPa) and (g) in portal venous phase (EPv), (h) long diameter (LD), (i) short diameter (SD), (j) the ratio of long diameter to short diameter (LD/SD), (k) contour (round; oval; irregular), (l) necrosis (yes or no), (m) calcification (yes or no), (n) surface ulceration (yes or no), (o) intratumoral angiogenesis (yes or no) and (p) peripheral enlarged lymph node (LN) (yes or no). The CT attenuation value was measured by drawing the region of interest (ROI) on the same axial slice of the tumor avoiding vessels, calcification, and the necrotic regions. DEAP or DEPP was obtained by subtracting CTU from CTA or CTV respectively. EPa or EPv was equal to DEAP or DEPP divided by CTU. Enlarged lymph node was considered present if the shortest axis diameter of lymph node was more than 10 mm. Some of the CT features referred to our previous report [27]. LD, SD, contour, necrosis, surface ulceration and intratumoral angiogenesis of tumors were the main aspects considered by radiologists when classifying tumors into low- or high-risk GISTs.

### Machine learning

Scikit-learn software was used to build three classifiers-DT, GBDT and LR. The detailed methodology is described on the website of official documentation (https://scikit-learn.org/), which has also been applied in our previous research [27]. The three datasets (training, internal validation and external test cohort) had no any intersection in our study. The training dataset aims to train the models, the internal validation cohort aims to adjust parameters and the external test cohort aims to evaluate the model performance. For each model, sensitivity, specificity, accuracy, positive predictive value (PPV), negative predictive value (NPV) and area under the curve (AUC) together with 95% confidence intervals (95% CI) were respectively calculated to assess the performance of each classifier. We observed a significant imbalance between the number of low-risk versus high-risk tumors in the three cohorts. The function of Class_Weight in scikit-learn soft was performed to solve the problem of unbalanced samples. In classification task, small sample categories (high-risk malignant) were assigned high weight and large sample categories (low-risk malignant) assigned low weight.

### Grid search strategy for selecting optimal parameters

In order to find the optimal parameters of three models, the grid search strategy in scikit-learn software was used. In the grid search process, 5-fold Cross-Validation (CV) was used to evaluate model performance. Meanwhile, the accuracy was used as an evaluation metric to maximize model performance. The detail of grid search method is described in the model selection module on the website of official documentation (https://scikit-learn.org/stable/model_selection.html#model-selection).

### Logistic regression (LR)

LR is the most conventional approach to measure the relationship between discrete response variables and several covariates by estimating probabilities. It can be written as: *p* = 1/(1 + e^− z^). z refers to logistic regression model. The response variable can take two values (0 as no and 1 as yes) according to *p* smaller than 0.5 or not.

The final optimal parameters of LR were set as follows: C = 100, random_state = 12, penalty = ’l1’, solver = ’liblinear’. Other parameter factors were set as default in sklearn software module.

### Decision tree (DT)

DT, as a binary method, can be used to classify data by calculating their characteristics. Decision nodes, branches and leaves are the three main components of DT. DT starts with a node and extends to many branches and child nodes, finally to leaves. The criterion used in our model is the Gini’s Diversity Index, which is a measure of node impurity. The standard CART algorithm, implemented using sciki-learn library in Python, was applied to build decision tree.

The parameters set in the DT were: random_state = 0, max_features = 6, max_depth = 6, criterion = ’gini’. Other parameters were set as default in sklearn software module.

### Gradient boosting decision tree (GBDT)

GBDT is an ensemble classifier based on bootstrap sampling, which aims to improve the generalization ability and robustness by combining the prediction results of multiple base learners (i.e. weak decision trees). The weight is adjusted with iteration, so that the higher weight will be assigned to the data poorly classified. Totally 15 weak decision trees were created in GBDT model in this study (e.g. a tree is showed in Fig S1).

The following parameter factors were used for GBDT: learning_rate = 0.1, max_depth = 8, random_state = 0, min_samples_leaf = 2. Other parameters were also set as default in the sklearn software.

### Performance comparison between radiologists and models

Diagnostic performance differences between the three ML models and the two radiologists were compared in the external test cohort. Before performance comparisons, intra-class correlation coefficients (ICCs) were calculated to assess agreement between the two reviewers.

### Feature variable analysis

GBDT and LR showed excellent diagnostic efficiency in predicting risk classification of gastric GISTs on account of the high accuracy and strong robustness. LR is well known for determining the beneficial features to support decision by linear analysis, since the result is easy to explain. Firstly, significant CT features were determined by univariate analysis. Secondly, variable with *P* less than 0.05 were as the input data to calculate the independent risk factors for high-risk malignant GISTs. In order to find out important features for high-grade malignant GISTs in GBDT, the function of Feature_Importance was performed. The description of feature importance is on the website: https://scikit-learn.org/stable/ modules/ensemble.html#gradient-tree-boosting. According to the official documentation description, individual decision trees in the GBDT model intrinsically performed feature selection by selecting appropriate split points. This information can be used to measure the importance of each feature. The basic idea is: the more often a feature is used in the split points of a tree, the more important that feature is. Subsequently, the feature variables of LR and GBDT were compared.

### Statistical analysis

P-P plots and Q-Q plots were used to assess normal distribution of data. Continuous distributed data were showed as mean ± SD, and categorical variables were expressed as n (%). Univariate analysis using t test or Mann-Whitney U test for continuous variables and Fisher’s exact test for categorical variables were performed to compare CT features between the low-grade malignancy and high-grade malignancy groups. Variables with *P* < 0.05 were considered as significant features and were included in the LR multivariate analysis. The final features with *P* < 0.05 from multivariate logistic regression model indicated the significant predictors of high risk GISTs. Statistical analyses were performed using SPSS version 22.0 (SPSS Inc., Chicago, IL, USA). A statistically significant difference was defined as two - sided *P* value < 0.05.

## Results

### Clinical characteristics of patients

231 patients (109 men and 122 women; mean age, 59.47 ± 10.13 years) from Center 1 and 78 patients (41 men and 37 women; mean age, 62.69 ± 10.78 years) from Center 2 were included in our series. The training cohort enrolled 161 patients with gastric GISTs consisting of 47 high-risk tumors and 114 low-risk ones. 70 patients with GISTs (21 high-risk tumors and 49 low-risk ones) and 78 patients with GISTs (16 high-risk tumors and 62 low-risk ones) constituted the internal validation cohort and the external test cohort, respectively.

Details of the clinical characteristics of three cohorts are shown in Table S1. Results of the univariate analysis indicated that patients in three databases had no significant difference in variables of age, sex, clinical symptom and tumor marker between the low-grade malignancy and the high-grade malignancy groups (all *P* > 0.05). The clinical characteristics of patients had no contribution to the prediction of malignancy in gastric GISTs.

### Univariate analysis of CT data

**Fig. 2:**
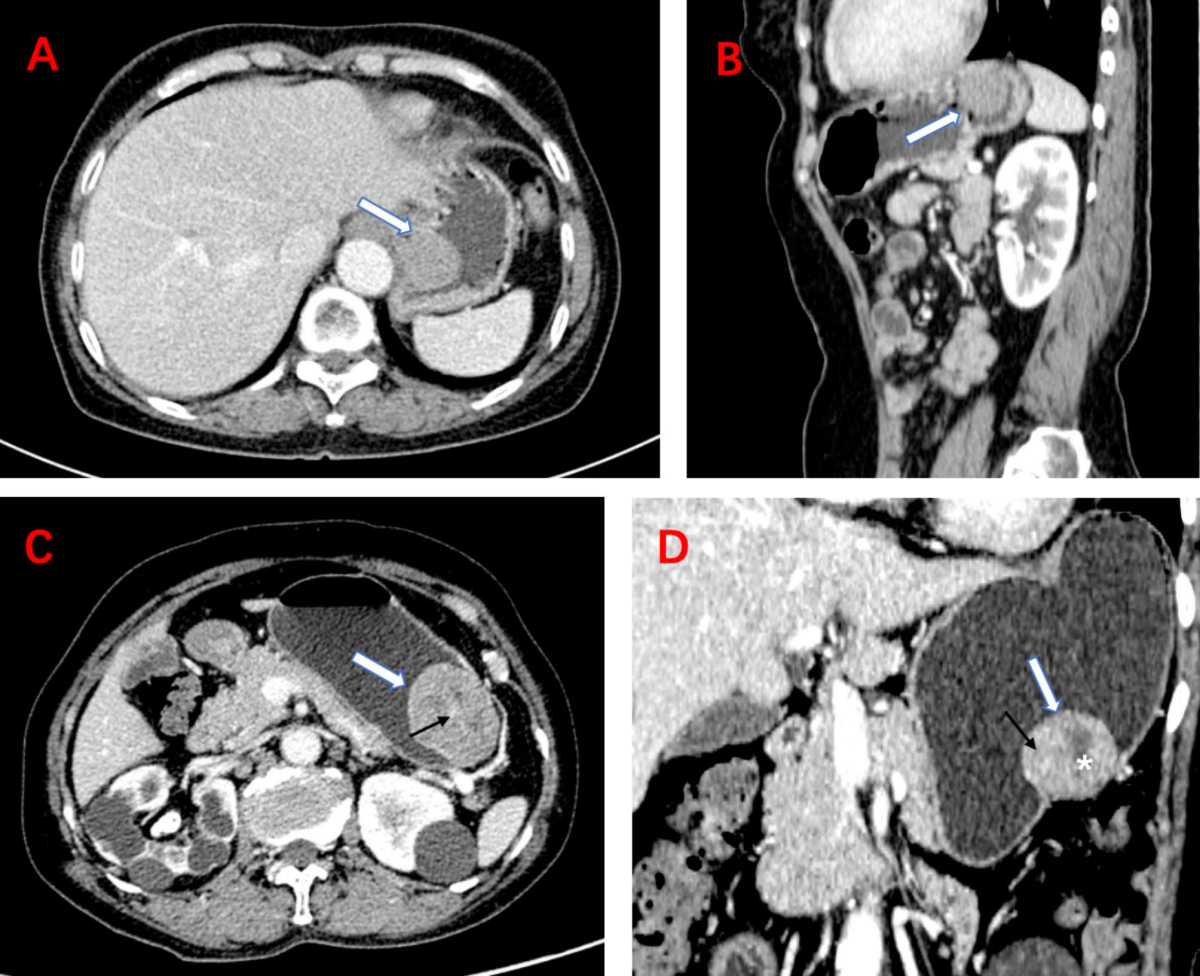
CT image of low-grade and high-grade malignant GISTs. (**A**-**B**). A low-grade malignant GIST in cardia in an elderly man. (**A**-**B**). Axial and sagittal CT scans in portal venous phase show a round mass with 2.5 cm of the long diameter (white arrows). The lesion has a homogeneous enhancement pattern without necrosis, calcification, and intratumoral angiogenesis in tumor. (**C**-**D**). A high-grade malignant GIST in gastric body in a middle-aged woman. Axial and coronal CT images in portal venous phase show an irregular neoplasm with 4.7 cm of the long diameter (white arrows). Intratumoral angiogenesis (black arrows) are seen in the lesion and the mass shows heterogeneous enhancement with necrotic portion (*) within the tumor

Results of univariate analysis of CT imaging features is exhibited in Table 1. LD, SD, contour, presence of necrosis and surface ulceration were showed to be significant features in distinguishing two groups in three cohorts. Size in high-grade malignancy GISTs was found larger than that of low-grade ones. Lesions with oval and irregular contours were seen more commonly in the high-grade malignancy group. Necrosis and surface ulceration were more likely to be found in the high-grade group. Intratumoral angiogenesis was significantly different between the two groups in the training and external test cohorts, but not in the internal validation cohort, as shown in Fig. 2. No difference of the remaining CT imaging variables was found in all three cohorts.

**Table 1:** Univariate analysis of CT features of GISTs in the training cohort, internal validation cohort and external test cohort

CT features	Training cohort (n = 161)			Internal validation cohort (n = 70)			External test cohort (n = 78)		
	Low-grade malignancy (n = 114)	High-grade malignancy (n = 47)	*P* value	Low-grade malignancy (n = 49)	High-grade malignancy (n = 21)	*P* value	Low-grade malignancy(n = 62)	High-grade malignancy(n = 16)	*P* value
**CTU(HU)**	33.90 ± 8.58	33.86 ± 6.38	0.976	34.87 ± 7.38	35.14 ± 6.08	0.882	36.60 ± 8.75	35.49 ± 5.27	0.577
**CTA(HU)**	53.24 ± 12.51	55.39 ± 15.60	0.359	58.11 ± 15.27	62.88 ± 12.49	0.212	58.52 ± 14.84	56.78 ± 14.26	0.617
**CTV(HU)**	67.38 ± 15.84	68.84 ± 15.11	0.592	72.2 ± 19.76	75.54 ± 16.24	0.499	75.03 ± 18.20	70.06 ± 18.58	0.296
**DEAP(HU)**	19.33 ± 11.15	21.53 ± 13.55	0.289	23.24 ± 13.33	27.74 ± 10.58	0.175	21.92 ± 15.65	21.29 ± 13.46	0.856
**DEPP(HU)**	34.47 ± 16.42	34.97 ± 13.55	0.582	37.34 ± 18.07	40.40 ± 16.33	0.507	38.43 ± 18.79	34.58 ± 18.23	0.440
**EPa**	0.65 ± 0.67	0.65 ± 0.39	0.971	0.69 ± 0.41	0.81 ± 0.34	0.245	0.67 ± 0.57	0.61 ± 0.42	0.694
**Epv**	1.14 ± 1.22	1.07 ± 0.44	0.685	1.11 ± 0.61	1.20 ± 0.57	0.560	1.14 ± 0.72	1.00 ± 0.58	0.455
**LD(mm)**	24.78 ± 10.39	33.81 ± 12.94	**0.000**	22.39 ± 10.27	37.43 ± 11.01	**0.000**	26.32 ± 10.53	43.56 ± 7.11	**0.000**
**SD(mm)**	20.43 ± 9.62	28.19 ± 10.65	**0.000**	18.98 ± 8.53	31.33 ± 9.46	**0.000**	22.06 ± 9.70	37.50 ± 6.84	**0.000**
**LD/SD**	1.24 ± 0.26	1.20 ± 0.16	0.286	1.18 ± 0.16	1.20 ± 0.15	0.576	1.23 ± 0.21	1.17 ± 0.12	0.120
**Contour**			**0.001**			**0.000**			**0.000**
Round	60(52.63%)	14(29.79%)		26(53.06%)	6(28.57%)		27(43.55%)	2(12.50%)	
Oval	38(33.33%)	15(31.91%)		19(38.78%)	3(14.29%)		24(38.71%)	4(25%)	
Irregular	16(14.04%)	18(38.30%)		4(8.16%)	12(57.14%)		11(14.74%)	10(62.50%)	
**Necrosis**	24(21.05%)	20(42.55%)	**0.007**	6(12.24%)	9(42.86%)	**0.011**	20(32.26%)	14(87.50%)	**0.000**
**Calcification**	12(10.53%)	4(8.51%)	0.921	7(14.29%)	1(4.77%)	0.461	10(16.13%)	3(18.75%)	1.000
**Surface ulceration**	10(8.77%)	10(21.28%)	**0.037**	1(2.04%)	8(38.10%)	**0.000**	5(8.06%)	5(31.25%)	**0.043**
**Intratumoral angiogenesis**	8(7.02%)	11(23.40%)	**0.006**	3(6.12%)	4(19.05%)	0.224	16(25.81%)	9(56.25%)	**0.032**
**LN**	0	1(2.13%)	0.295	0	1(4.77%)	0.300	0	1(6.25%)	0.208

CTU/CTA/CTV, the CT attenuation value in unenhancement phase/arterial phase/venous phase; DEAP/ DEPP, degree of enhancement in arterial phase/venous phase; EPa/EPv, enhanced potentiality in arterial phase/venous phase; LD, long diameter; SD, short diameter; LN, peripheral enlarged lymph nodes

*P* values written in bold indicate significant difference between low-grade malignancy and high-grade malignancy groups

### Model evaluation

**Fig. 3:**
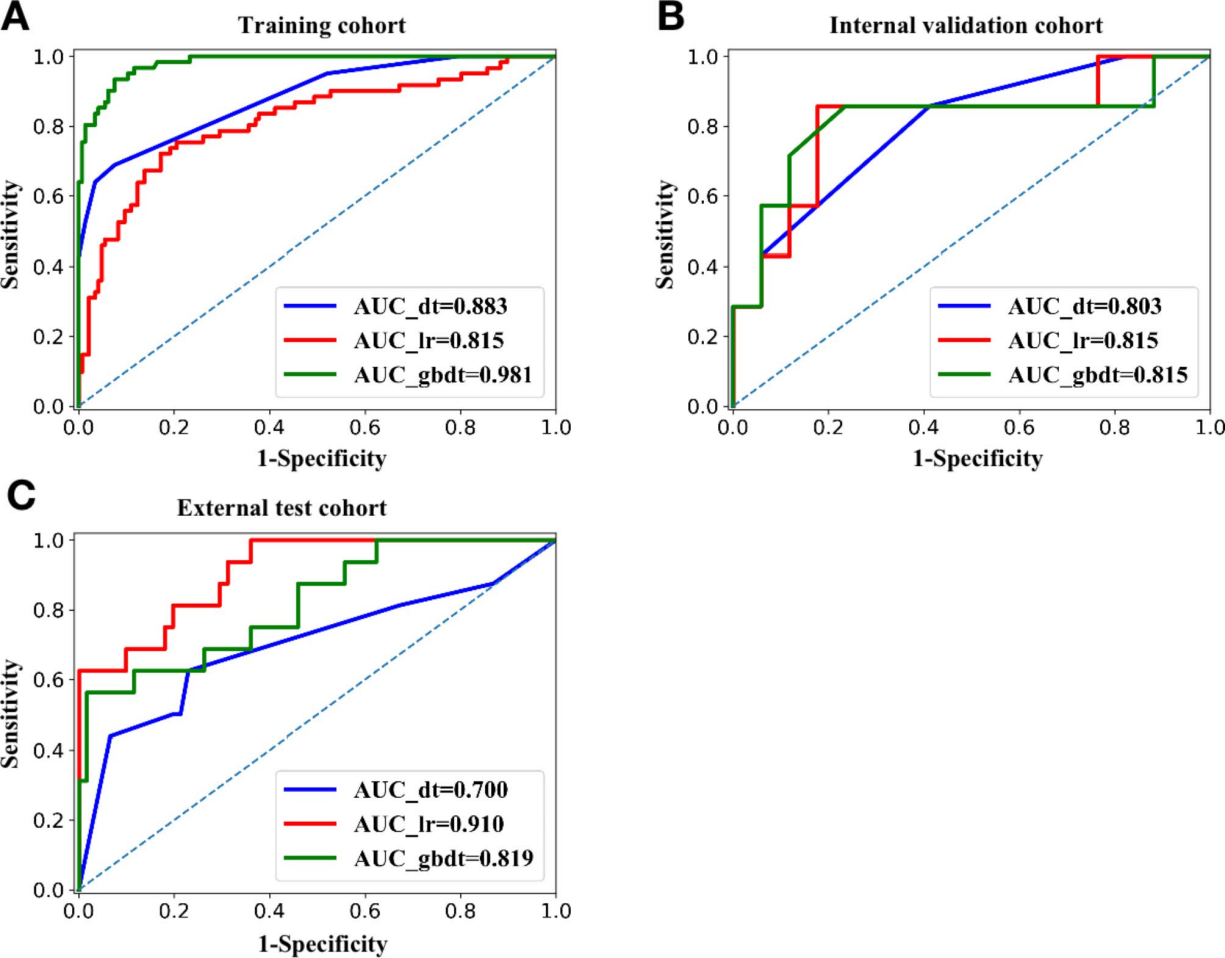
Receiver operating characteristic (ROC) curves of three models to predict the risk stratification of gastric GISTs. (**A**). ROC curve of three models in the training cohort. The largest area under the curve (AUC) was GBDT (0.981), followed by DT (0.883) and LR (0.815). (**B**). ROC curve of three models in the internal validation cohort. GBDT and LR had the equal AUC (0.815), and DT gained the smallest AUC (0.803). (**C**). ROC curve of three models in the external test cohort. LR gained the best AUC (0.910), followed by GBDT (0.819) and DT (0.700)

**Fig. 4:**
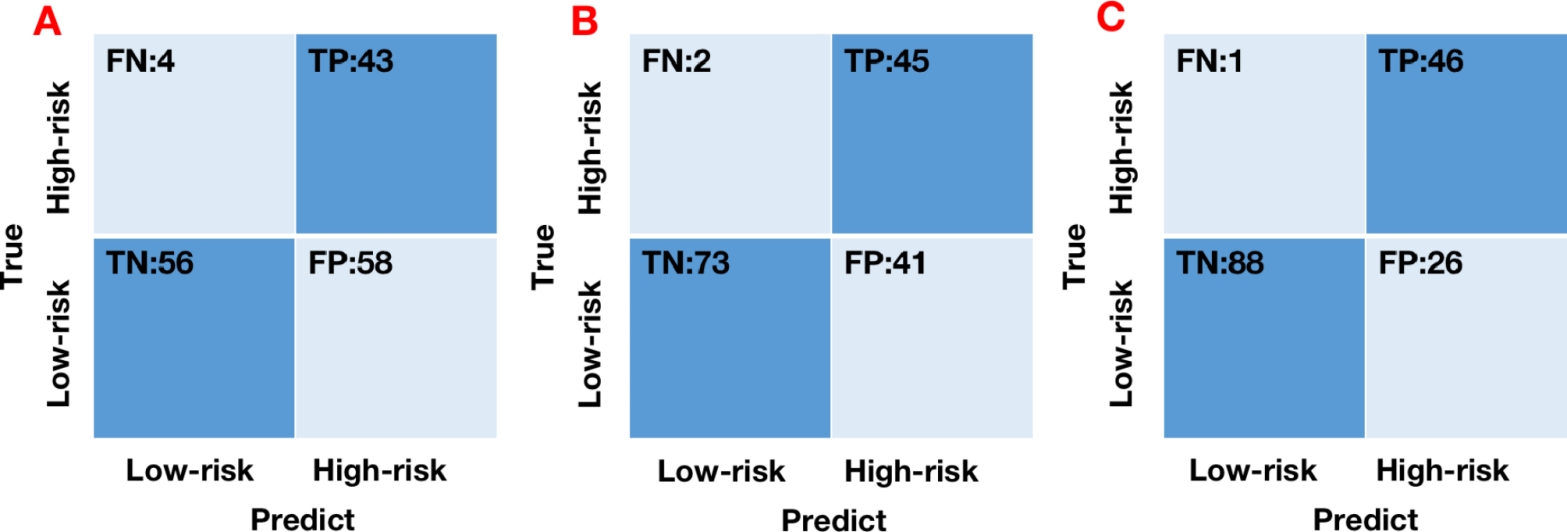
Confusion matrixes of LR (**A**), DT (**B**) and GBDT (**C**) models in the training cohort

Results of diagnostic performance of LR, DT and GBDT are described in Table 2; Fig. 3. GBDT gained the largest sensitivity (0.986), specificity (0.770), accuracy (0.923), PPV (0.639), NPV (0.993) and AUC (0.981) among the three models in the training cohort. Due to the compromise between sensitivity and specificity, accuracy and AUC are considered as better diagnostic indicators. GBDT achieved the largest AUC (0.981 and 0.815) among all three classifiers in the training and internal validation cohorts. Nevertheless, we found that LR had the largest AUC (0.910), followed by GBDT (0.819) and DT (0.700) in the external test cohort. The lowest accuracy (0.790 and 0.727) and AUC values (0.803 and 0.700) were gained in DT model both in the internal validation cohort and the external test cohort. GBDT and LR performed best among the three models and in three cohorts with high accuracy and strong robustness. Confusion matrixes of three models in the training cohort are showed in Fig. 4. Figure S2 and S3 exhibit confusion matrixes of three models in the internal validation cohort and external test cohort, respectively. We also tried to build the stepwise logistic regression models using all CT features or significant features by univariate analysis in the training cohort. The accuracy and AUC together with 95%CI of stepwise logistic regression model using all CT features were 0.789 and 0.770 (0.668–0.860), slightly less than those of LR model built with independent risk factor. In addition, stepwise logistic regression model leaving six significant CT features by univariate analysis yielded 0.727 of accuracy and 0.732 (0.648–0.846) of AUC together with 95%CI, less than those of LR model built in this study as well. Results are showed in Figure S4.

**Table 2:** Diagnostic performance analysis of LR, DT and GBDT models

Classifier	Group	Sensitivity (95%CI)	Specificity (95%CI)	Accuracy (95%CI)	PPV	NPV	AUC (95%CI)
LR	Training cohort	0.918(0.839–0.996)	0.492(0.400-0.584)	0.792(0.729–0.855)	0.426	0.933	0.815 (0.744–0.885)
	Internal validation cohort	0.941(0.840-1.000)	0.437(0.312–0.576)	0.792(0.744–0.841)	0.417	0.955	0.815 (0.602–0.904)
	External test cohort	0.852(0.678–0.999)	0.688(0.573–0.803)	0.818(0.732–0.964)	0.424	0.956	0.910 (0.810–0.978)
DT	Training cohort	0.966(0.914–0.997)	0.639(0.551–0.727)	0.870(0.818–0.922)	0.525	0.979	0.883 (0.826–0.941)
	Internal validation cohort	0.941(0.840–0.996)	0.429(0.290–0.568)	0.790(0.695–0.885)	0.414	0.944	0.803 (0.587–0.845)
	External test cohort	0.787(0.586–0.988)	0.500(0.376–0.625)	0.727(0.628–0.826)	0.289	0.901	0.700 (0.545–0.856)
GBDT	Training cohort	0.986(0.952–0.997)	0.770(0.693–0.847)	0.923(0.882–0.964)	0.639	0.993	0.981 (0.957-1.000)
	Internal validation cohort	0.882(0.744-1.000)	0.714(0.588–0.841)	0.833(0.746–0.920)	0.570	0.934	0.815 (0.704–0.920)
	External test cohort	0.918(0.784–0.999)	0.563(0.442–0.687)	0.844(0.764–0.925)	0.352	0.964	0.819 (0.686–0.952)

LR, Logistic regression; DT, Decision tree; GBDT, Gradient boosting decision tree; AUC, area under the curve; CI, confidence interval, NPV, negative predictive value; PPV, positive predictive value

### Performance comparison between radiologists and models

ICC of 0.83 indicated that the agreement between two radiologists was good. Table 3 displays the two reviewers’ diagnostic performance in the external test cohort. GBDT and LR showed more favorable performances than two radiologists.

**Table 3:** Results of radiologists’ diagnostic performance in the external test cohort

	Sensitivity(95%CI)	Specificity(95%CI)	Accuracy(95%CI)	PPV	NPV	AUC (95%CI)
Reviewer 1	0.625(0.388–0.862)	0.484(0.360–0.608)	0.645(0.539–0.751)	0.313	0.833	0.628 (0.561–0.785)
Reviewer 2	0.750(0.538–0.962)	0.565(0.442–0.688)	0.758(0.663–0.853)	0.308	0.897	0.764 (0.603–0.912)

AUC, area under the curve; CI, confidence interval, NPV, negative predictive value; PPV, positive predictive value

### Feature variable analysis

**Fig. 5:**
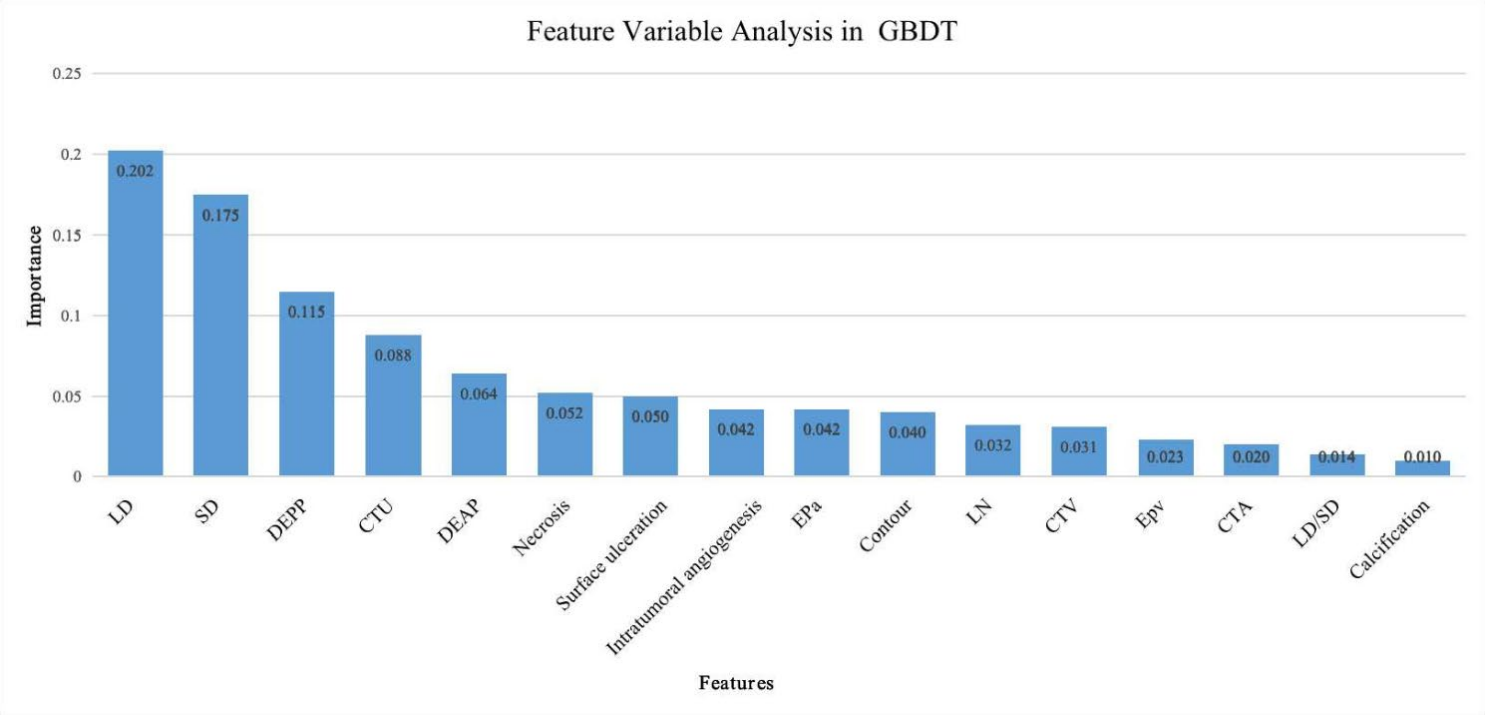
Features importance rank in GBDT model. Five top important features were as follows: LD (importance score 0.202), SD (0.175), DEPP (0.115), CTU (0.088) and DEAP (0.064). The remaining features had low importance scores

LD, SD, contour, necrosis, surface ulceration and intratumoral angiogenesis were selected as significant features by univariate analysis to input into multivariate analysis. Table 4 shows that only LD is an independent risk factor for high-grade malignant GISTs (*P* < 0.001, OR = 1.066). Results of important characteristics rank in GBDT are reported in Fig. 5. LD ranked the most important feature among all CT features with importance score of 0.202, followed by SD (0.175), DEPP (0.115), CTU (0.088) and DEAP (0.064). The remaining features had low importance scores. LD was demonstrated as the only same and most important feature for LR and GBDT in terms of feature variable analysis.

**Table 4:** Results of feature variable analysis in LR model

CT features	β	OR	OR (95%CI)	*P* value
LD	0.064	1.066	1.034–1.099	0.000
SD				0.550
Contour				0.288
Necrosis				0.658
Surface ulceration				0.404
Intratumoral angiogenesis				0.248

LD, long diameter; SD, short diameter; OR, odds ratio; CI, confidence interval

*P* values written in bold indicate significant difference

## Discussion

To our best knowledge, this is the first research on the prediction of malignancy in gastric GISTs by machine learning classifiers. In addition, our report focuses on GISTs tumors of 1–5 cm in the gastric, which is different from studies that include large-size GISTs located in various sites of the gastrointestinal tract. This study has the largest sample size among relevant studies, so the reliability of the results can be guaranteed. Various qualitative and quantitative variables extracted from CT signs were inputted into LR, DT and GBDT models. The results of model evaluation were different but not inconsistent among the three cohorts. For the training cohort, GBDT had the greatest sensitivity, specificity, accuracy, PPV, NPV and AUC among the three models. What’s more, GBDT gained the largest AUC in the training and internal validation sets and performed best in all three cohorts in terms of accuracy, although the AUC was not as good as LR in the external test cohort. Furthermore, GBDT and LR showed better performance than the two radiologists. However, the performance of DT was not as outstanding as GBDT and LR. Therefore, GBDT and LR were suggested to be promising ML models for CT-based risk classification prediction of gastric GISTs due to the high accuracy and strong robustness.

GBDT, an ensemble method based on bootstrap sampling, was demonstrated to be a favorable algorithm with high predictive efficiency, as reported in various previous researches [28–31]. The excellent performance of GBDT classifier is attributed to its ability to optimize the algorithm by increasing the weight of weak decision trees. As a classic ML algorithm for solving two-class classification problem, LR assumes that the data conform to the Bernoulli distribution, and then calculates the parameters through the maximum likelihood function method to achieve two classification. Additionally, probability prediction obtained from LR model can be preferentially utilized to better assist in decision making. However, LR has some limitations. First, logistic regression analysis, which is difficult to fit the true distribution of data, has only linear decision boundary. Second, it may weaken the performance of classification task due to the limitation of overfitting and multicollinearity. In terms of DT, a tree consisting of decision nodes, branches, and leaves, is generated using the training dataset, and the test dataset is classified or predicted. In this study, the ability of DT in predicting the risk grading of gastric GISTs may be weaker than LR and GBDT.

When it comes to feature variable selection, LD was found to be the only common CT feature between LR and GBDT that distinguished for high-grade malignant GISTs in this study. Several studies using multivariate logistic regression analysis revealed that the size of GISTs was the only independent risk factor for differentiation of the high-grade malignant GISTs [16, 21], so did the gastric GISTs with size shorter than 5 cm in our study. Kim et al. [21] reported that for GISTs ≤ 5 cm, it was not possible to identify malignant from benign by tumor size based on CT scan images, which contradicted with our results. It may be related to different grouping definitions and different tumor composition ratios. Mazzei et al. [32] found that the maximum diameter of GISTs with high mitotic index (> 5 mitoses) was larger than that of GISTs with low mitotic index (≤ 5 mitoses), suggesting that the larger the tumor was, the faster it grew and the the higher degree of malignancy it had. However, oval and irregular contours, the presence of necrosis, surface ulceration and intratumoral angiogenesis appeared more frequently in the high-grade malignancy group, similar to other studies [17, 19, 21, 22], however, these features were excluded from the selection of predictors in LR. GBDT algorithm could determine complicated and impalpable feature relationships to support decision-making that may not be detected in logistic regression analysis [33]. In this study, DEPP, CTU and DEAP were selected as important features despite not being significant different factors in univariate analysis. The important features that GBDT concentrated on appear to be unimportant features in traditional statistical method, from which, high predictive performance can be obtained.

Artificially determined CT imaging features were used as the input variables for the three ML models to predict the risk classification of gastric GISTs in this study, and great results were obtained, especially in GBDT and LR. Compared with the diagnostic ability of radiologists, ML achieved more promising results, which may have a guiding prospect for doctors in daily diagnostic work, especially for the junior ones. It may promisingly provide theoretical and practical support for texture analysis or deep learning since ML may play an important role in feature selection.

There are some limitations in our study. First, our sample size was small for ML. ML classifiers can highlight their advantages in the context of large data, amounts of predictor variables or complex relationship. Second, four risk grades were finally divided into two, so the results were unable to meet the requirement of each risk classification. Simple ML model cannot meet the needs of predicting four risk levels, but the convolutional neural network can, which puts the next step of research on the agenda. Third, only three simple ML models were implemented in our research, including the classic LR. We will try other more complex ML models to assess the risk stratification, such as random forest, support vector machine, k-nearest neighbors, etc. Fourth, radiomics, which transforms medical images into high-dimensional data by extracting tumor’s shape, intensity, and texture features, has recently shown great potential in aiding clinical decision making. Developing CT-based radiomics models for GIST risk stratifcation will be a future work.

## Conclusions

In summary, GBDT and LR showed outstanding performance with high accuracy and strong robustness in the risk assessment of gastric GISTs less than 5 cm on CT imaging. The long diameter of lesion was found to be the most important feature for risk stratification.

## Electronic supplementary material

Below is the link to the electronic supplementary material.

Supplementary Material 1

Supplementary Material 2

## Data Availability

All datasets presented and analyzed in this study were interpreted and provided by the corresponding author.

## Abbreviations

AUC: Area under the curve
CE-CT: Contrast-enhanced CT
CSCO: Chinese Society of Clinical Oncology
CTU/CTA/CTV: CT attenuation value in unenhancement phase/arterial phase /venous phase
CV: Cross-Validation
DEAP/ DEPP: Degree of enhancement in arterial phase/venous phase
DT: Decision tree
EPa/EPv: Enhanced potentiality in arterial phase/venous phase
GBDT: Gradient boosting decision tree
GISTs: Gastric gastrointestinal stromal tumors
HU: Hounsfield unit
ICC: Intra-class correlation coefficient
LD: Long diameter
LN: Peripheral enlarged lymph nodes
LR: Logistic regression
ML: Machine learning
NCCN: National Comprehensive Cancer Network
NPV: Negative predictive value
PPV: Positive predictive value
SD: Short diameter
