# Supplementary material for: Machine learning for predicting the risk stratification of 1–5 cm gastric gastrointestinal stromal tumors based on CT
Source: BMC Med Imaging. 2023 Jul 6;23:90. doi: 10.1186/s12880-023-01053-y (PMC10327391; doi:10.1186/s12880-023-01053-y)
Supplement: Supplementary file 1 — Supplementary Material 1 [file 12880_2023_1053_MOESM1_ESM.docx]

**FIGURE S1**
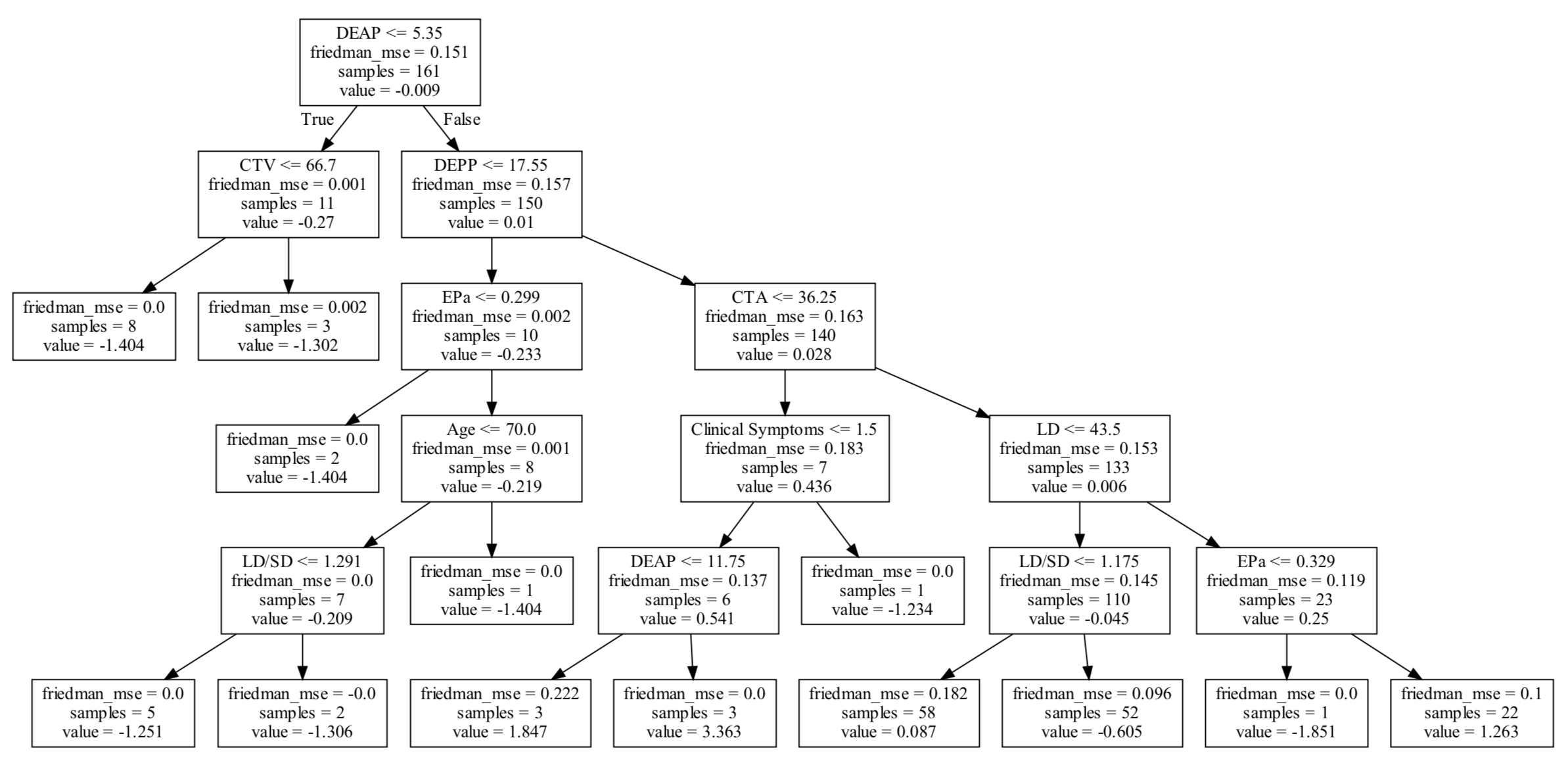


**Figure S1.** A tree in GBDT classifier.

**FIGURE S2**

**
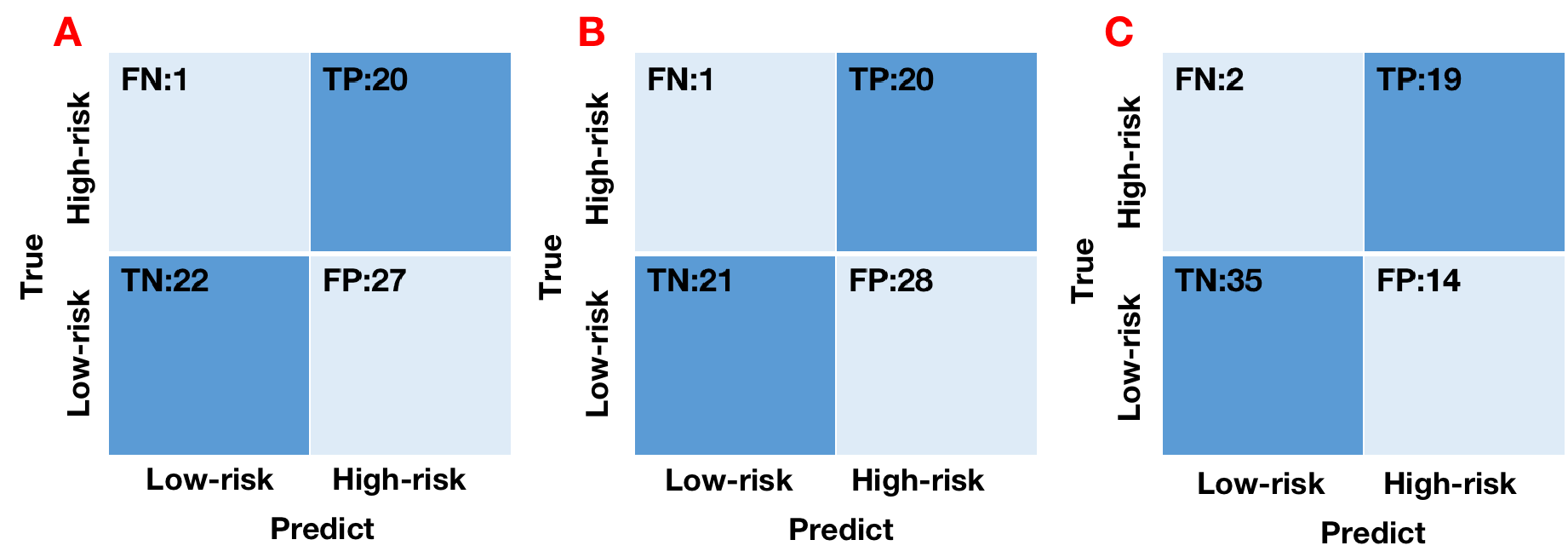
**

**Figure S2.** Confusion matrixes of LR (A), DT(B) and GBDT (C) models in the internal validation cohort.

**FIGURE S3**

**
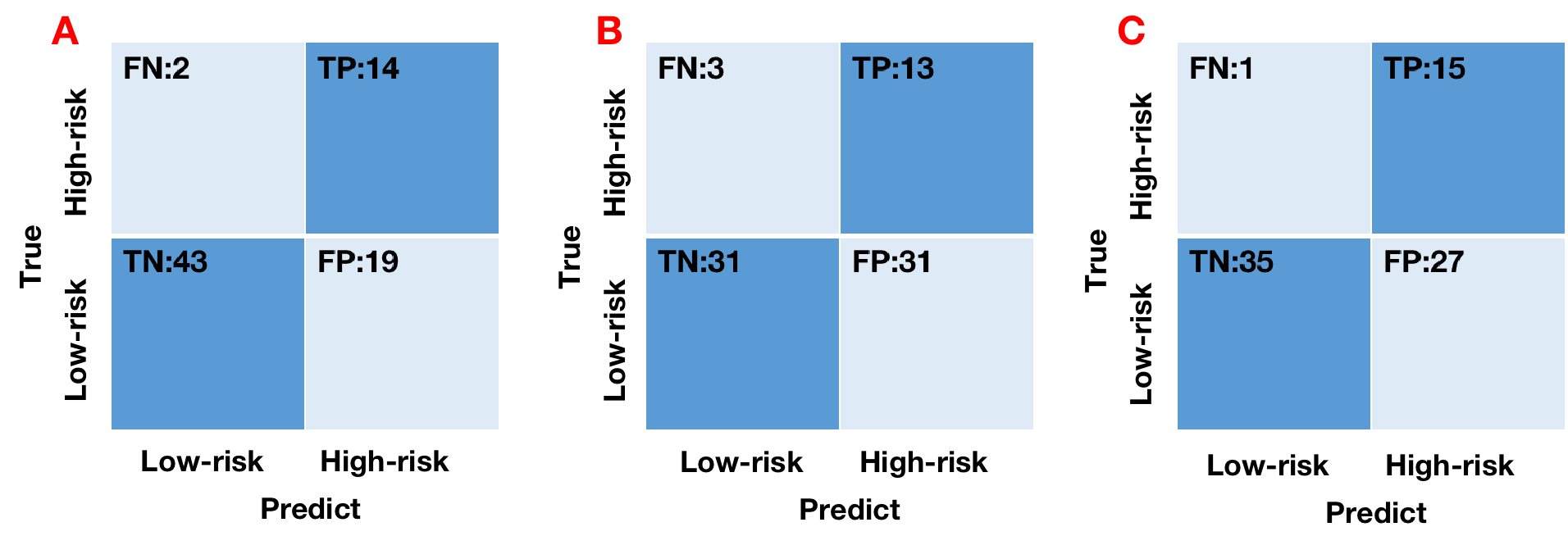
**

**Figure S3.** Confusion matrixes of LR (A), DT(B) and GBDT (C) models in the external test cohort.

**
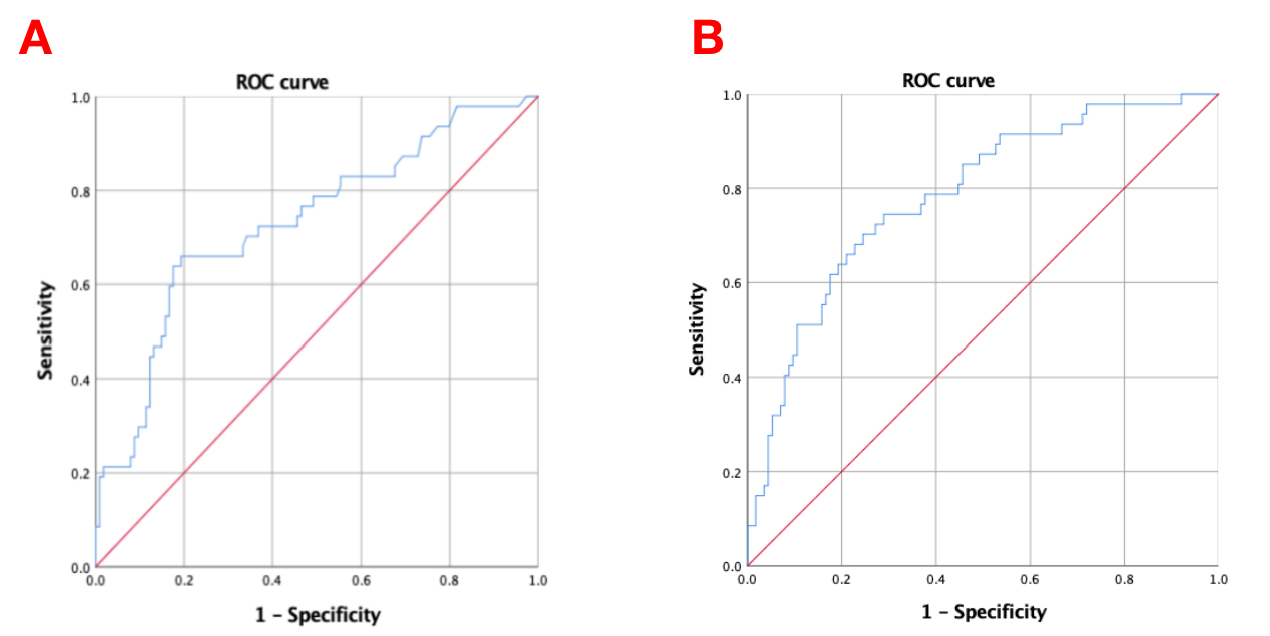
**

**Figure S4.** Receiver operating characteristic (ROC) curves of LR model using six significant features by univariate analysis (A) and LR model using all CT features(B) in the training cohort.
