# Supplementary material for: Machine learning for predicting the risk stratification of 1–5 cm gastric gastrointestinal stromal tumors based on CT
Source: BMC Med Imaging. 2023 Jul 6;23:90. doi: 10.1186/s12880-023-01053-y (PMC10327391; doi:10.1186/s12880-023-01053-y)
Supplement: Supplementary file 2 — Supplementary Material 2 [file 12880_2023_1053_MOESM2_ESM.docx]

**TABLE S1.** Clinical characteristics of GISTs in the training cohort, internal validation cohort and external test cohort.

| **Clinical characteristics** | **Training cohort (n=161)** | | | **Internal validation cohort (n=70)** | | | **External test cohort (n=78)** | | |
| --- | --- | --- | --- | --- | --- | --- | --- | --- | --- |
|  | **Low-grade malignancy**  **(n=114)** | **High-grade malignancy**  **(n=47)** | ***P* value** | **Low-grade malignancy**  **(n=49)** | **High-grade malignancy**  **(n=21)** | ***P* value** | **Low-grade malignancy**  **(n=62)** | **High-grade malignancy (n=16)** | ***P* value** |
| **Age, mean ± SD (years)** | 60.25±9.89 | 59.60±10.16 | 0.704 | 58.27±10.28 | 57.71±11.69 | 0.844 | 62.15±10.69 | 64.81±11.20 | 0.394 |
| **Sex** |  |  | 0.298 |  |  | 1.000 |  |  | 0.783 |
| Male | 58(50.88%) | 19(40.43%) |  | 22(44.90%) | 10(47.62%) |  | 30(48.39%) | 7(43.75%) |  |
| Female | 56(39.12%) | 28(59.57%) |  | 27(55.10%) | 11(52.38%) |  | 32(51.61%) | 9(56.25%) |  |
| **Symptom** |  |  | 0.225 |  |  | 0.511 |  |  | 0.927 |
| Yes | 80(70.18%) | 28(59.57%) |  | 29(59.18%) | 15(71.43%) |  | 34(54.84%) | 8(50%) |  |
| No | 34(29.82%) | 19(40.43%) |  | 20(40.82%) | 6(28.57%) |  | 28(45.16%) | 8(50%) |  |
| **Tumor marker** |  |  | 0.260 |  |  | 1.000 |  |  | 0.880 |
| Yes | 38(33.33%) | 16(34.04%) |  | 14(28.57%) | 6(28.57%) |  | 12(19.35%) | 2(12.50%) |  |
| No | 76(66.67%) | 31(65.96%) |  | 35(71.43%) | 15(71.43%) |  | 50(80.65%) | 14(87.50%) |  |

GISTs, gastrointestinal stromal tumors.
